# The SIM Time Network

**DOI:** 10.6028/jres.116.005

**Published:** 2011-04-01

**Authors:** Michael A. Lombardi, Andrew N. Novick, J. Mauricio Lopez R, Francisco Jimenez, Eduardo de Carlos Lopez, Jean-Simon Boulanger, Raymond Pelletier, Ricardo J. de Carvalho, Raul Solis, Harold Sanchez, Carlos Andres Quevedo, Gregory Pascoe, Daniel Perez, Eduardo Bances, Leonardo Trigo, Victor Masi, Henry Postigo, Anthony Questelles, Anselm Gittens

**Affiliations:** National Institute of Standards and Technology (NIST), Boulder, CO 80305, USA; Centro Nacional de Metrologia (CENAM), Querétaro, Mexico; National Research Council (NRC), Ottawa, Canada; National Observatory (ONRJ), Rio de Janeiro, Brazil; Centro Nacional de Metrologia de Panama (CENAMEP), Panama City, Panama; Instituto Costarricense de Electricidad (ICE), San Jose, Costa Rica; Superintendencia de Industria y Comercio (SIC), Bogota, Colombia; Bureau of Standards Jamaica (BSJ), Kingston, Jamaica; Instituto Nacional de Tecnologia Industrial (INTI), Buenos Aires, Argentina; Laboratorio Nacional de Metrologia (LNM), Guatemala City, Guatemala; Administracion Nacional De Usinas Y Trasmisiones Electricas (UTE), Montevideo, Uruguay; Instituto Nacional de Tecnologia Normalizacion y Metrologia (INTN), Asuncion, Paraguay; Servicio Nacional de Metrologia (SNM), INDECOPI, Lima, Peru; Trinidad and Tobago Bureau of Standards (TTBS), Trinidad and Tobago; Saint Lucia Bureau of Standards (SLBS), Castries, Saint Lucia

**Keywords:** frequency, Internet, regional metrology organization, time, traceability

## Abstract

The *Sistema Interamericano de Metrologia* (SIM) is a regional metrology organization (RMO) whose members are the national metrology institutes (NMIs) located in the 34 nations of the Organization of American States (OAS). The SIM/OAS region extends throughout North, Central, and South America and the Caribbean Islands. About half of the SIM NMIs maintain national standards of time and frequency and must participate in international comparisons in order to establish metrological traceability to the International System (SI) of units. The SIM time network (SIMTN) was developed as a practical, cost effective, and technically sound way to automate these comparisons.

The SIMTN continuously compares the time standards of SIM NMIs and produces measurement results in near real-time by utilizing the Internet and the Global Positioning System (GPS). Fifteen SIM NMIs have joined the network as of December 2010. This paper provides a brief overview of SIM and a technical description of the SIMTN. It presents international comparison results and examines the measurement uncertainties. It also discusses the metrological benefits that the network provides to its participants.

## 1. Introduction to SIM

The goal of the *Sistema Interamericano de Metrologia* (SIM) is to ensure the uniformity of measurements throughout its region. SIM metrology working groups pursue this goal by collaborating on training programs and technical projects, and by reviewing the quality systems and calibration and measurement capabilities (CMCs) of the NMIs. They also organize inter-laboratory comparisons. These comparisons help NMIs establish traceability and maintain standards that are accurate enough to support their nation’s economy.

**Fig. 1:**
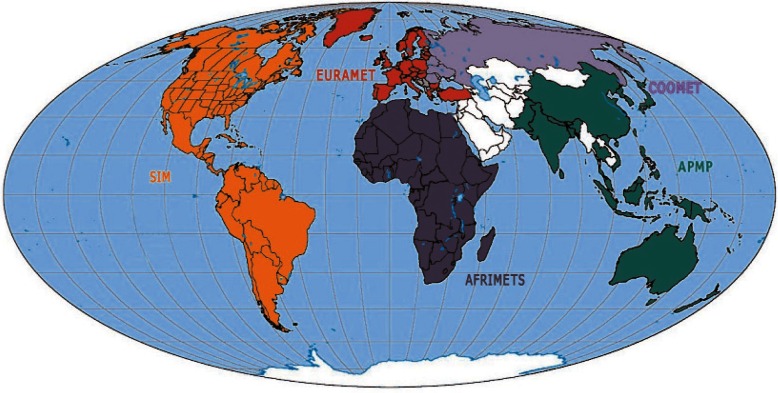
The world’s regional metrology organizations.

Each RMO faces its own unique challenges, and SIM faces several. SIM is the largest RMO in terms of land area (Fig. 1), and there is a large variation in both the populations of the SIM nations and the strength of their economies. The SIM region extends throughout North, Central, and South America and the Caribbean, an area that encompasses roughly 27 % of the world’s land mass and some 13 % of its population (an estimated 910 million people as of 2009). However, as of 2009, about two-thirds of the SIM population (approximately 617 million people) reside in the United States, Brazil, or Mexico. In contrast, 11 SIM nations, mostly islands in the Caribbean region, have populations of less than one million. As of 2009, the per capita gross domestic product (GDP) of the United States and Canada exceeded $38 000 USD, but 15 SIM nations had per capita GDPs of less than $10 000 USD [1]. This disparity in population and money directly translates into the level of resources that are made available for metrology. For example, NIST has about 40 full-time professionals employed in its time and frequency division, but many SIM NMIs are fortunate if even one metrologist is free to focus on time and frequency measurements.

## 2. History and Design Goals of the SIMTN

Informal discussions about a SIM Time Network (SIMTN) began at the National Institute of Standards and Technology (NIST) in the United States in 2003, but the plans to move ahead with development were not formalized until July 2004, at a meeting held at the National Research Council (NRC) in Canada. This meeting was attended by representatives of the three North American NMIs: the Centro Nacional de Metrología (CENAM) of Mexico, NRC, and NIST of the United States. At the time of this meeting, cooperation in time and frequency within the SIM region had essentially been limited to North America. NRC and NIST already had long standing reputations as internationally recognized timing laboratories, and CENAM (an NMI formed in 1994) had made rapid progress. With the exception of the National Observatory Rio de Janeiro (ONRJ) in Brazil, the other NMIs in the SIM region were not well known in the international time and frequency community and had little previous interaction with NIST, NRC, or CENAM.

The discussions in Canada focused on linking the NMIs of the Americas together, so that as many NMIs as possible could establish measurement traceability to the SI. This “linking” had to allow for the varying levels of resources of the laboratories and the different obstacles that they face. The North American NMIs, ONRJ, and the Centro Nacional de Metrología de Panamá (CENAMEP) in Panama already participated in the Bureau International des Poids et Mesures (BIPM) key comparisons. However, not all SIM NMIs had signed the BIPM Mutual Recognition Agreement (MRA), and some lacked the resources, training, experience, and contacts that participation in the BIPM key comparisons require. What was needed was a new mechanism for international comparisons that had as few barriers to entry as possible. The discussions resulted in a decision to build a time network that met the following design goals:

- To build a network that allowed all SIM NMIs to compare their time standards to those of the rest of the world.
- To utilize equipment that was low cost and easy to install, operate, and use, because SIM NMIs typically have small staffs and limited resources.
- To be capable of measuring the best standards in the SIM region. This meant that the measurement uncertainties had to be as small, or nearly as small, as those of the BIPM key comparisons.
- To report measurement results in near real-time, without the processing delays of the BIPM key comparisons.
- To build a democratic network that favored no single laboratory or nation, and to allow all members to view the results of all comparisons.

Once these design goals were established, the development of the network quickly proceeded. SIM measurement systems were delivered by NIST to CENAM and NRC in the spring of 2005, and the first SIMTN comparisons began in May of that same year [2].

## 3. Technical Description of the SIMTN

The SIMTN is based on common-view observations of the Coarse / Acquisition (C / A) codes transmitted by GPS satellites on the L1 carrier frequency of 1575.42 MHz. This technique was used to compare remote clocks shortly after the first GPS satellite was launched into orbit [3] and is one of several techniques used to derive Coordinated Universal Time (UTC) [4].

The common-view method [5] is simple but effective. The best possible comparison between two clocks would involve bringing both clocks to the same location. However, when the two clocks are not at the same location, the time difference between them can still be measured by simultaneously comparing both clocks to a signal in “common-view” of both sites. The difference between the two comparisons reveals the time difference between the two clocks. The common-view signal is simply a vehicle used to transfer time from one location to another.

**Fig. 2:**
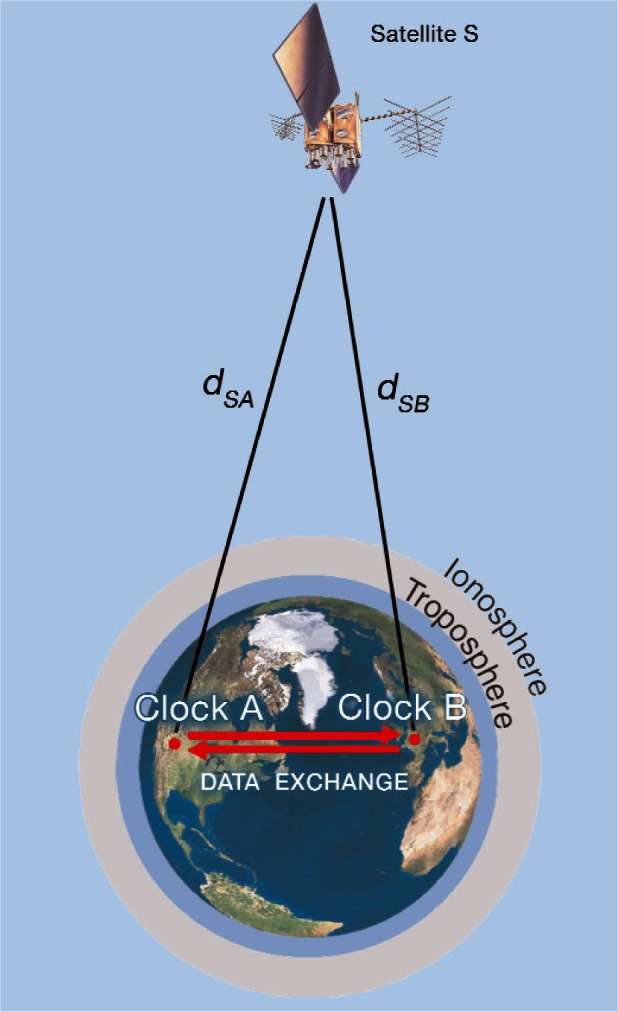
Common-view GPS measurements.

When GPS is used, the method involves a GPS satellite (*S*), and two receiving sites (*A* and *B*), each containing a GPS receiver and a local clock (Fig. 2). The GPS satellite transmits a signal that is received at sites *A* and *B*, and both sites compare the GPS signal to their local clock. Site A receives GPS over the path *d_SA_* and measures *Clock A* – *S*. Site *B* receives GPS over the path *d_SB_* and measures *Clock B* – *S*.

The difference between the two measurements is an estimate of *Clock A* – *Clock B*. Delays that are common to both paths *d_SA_* and *d_SB_* cancel even if they are unknown, but uncorrected delay differences between the two paths add uncertainty to the measurement result. Thus, the basic equation for a CVGPS measurement is

(1) $$(\mathrm{Clock}\;A-S)-(\mathrm{Clock}\;B-S)=(\mathrm{Clock}\;A-\mathrm{Clock}\;B)+(e_{\mathrm{SA}}-e_{\mathrm{SB}}).$$

The components that make up the (*e_SA_* − *e_SB_*) error term include delay differences between the two sites caused by ionospheric and tropospheric delays, multipath signal reflections, environmental conditions, and errors in the GPS antenna coordinates. These factors can be measured or estimated and either applied as a correction to the measurement or accounted for in the uncertainty analysis.

In its default configuration, the SIMTN implements the “classic” common-view technique. This technique aligns and differences data from the individual satellite tracks, and discards data collected from satellites that are not in common view at both sites. The average time difference, *TD*, between the clocks at the two sites is obtained by:

(2) $$\mathrm{TD}=\frac{\sum_{i=1}^{N}\left({\mathrm{REFGPS}}_{i}(A)-{\mathrm{REFGPS}}_{i}(B)\right)}{N},$$

where *N* is the number of satellites tracked at both sites, *REFGPS_i_*(*A*) is the series of individual satellite tracks recorded at site A, and *REFGPS_i_*(*B*) is the series of tracks recorded at site B. However, “classic” common-view does not always work across the wide geographic area covered by the SIMTN, because there are intervals when no satellites are in common view at both sites. For example, for the 8623.5 km baseline between NIST and ONRJ there are no satellites in common-view about 10 % of the time, and on average, only 1.4 satellites are simultaneously visible at both sites [6]. To allow for these situations, the SIM network can also present results using the “all-in-view” method where the satellite tracks are not aligned and no tracks are discarded. Instead, the averages of the *REFGPS_i_*(*A*) and *REFGPS_i_*(*B*) data series are calculated, and the time difference *TD* is simply the difference between the two averages:

(3) $$\mathrm{TD}=\bar{{\mathrm{REFGPS}}_{i}(A)}-\bar{{\mathrm{REFGPS}}_{i}(B)}.$$

A variation of the all-in-view technique has been used by the BIPM since September 2006 to process the GPS data used in the calculation of UTC [7]. The all-in-view method can provide slightly better results when the length of the baseline exceeds 5000 km, but its main advantage is that it can always be used, even when no satellites are in common view. This allows comparisons to be made between two clocks located anywhere on Earth.

**Fig. 3:**
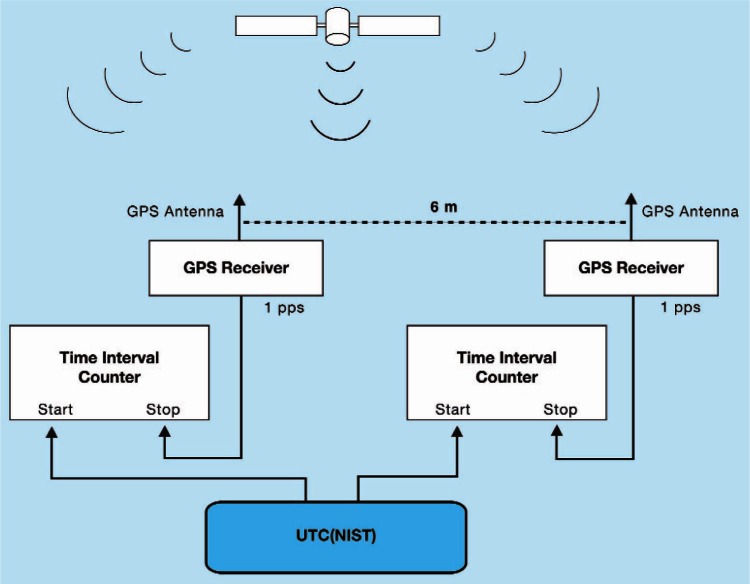
The common-clock calibration method.

To minimize the size of the (*e_SA_* − *e_SB_*) error term, all SIM systems are calibrated at NIST prior to shipment to the host NMI. Each calibration lasts for 10 days and is performed by use of the common-clock method (Fig. 3) across a 6 m baseline. A calibration is accepted only if there are no signal outages or equipment interruptions during the 10-day period, and if the time deviation (TDEV) of the common-clock comparison is near 0.2 ns at τ = 1 day. The calibration results in a single delay constant that accounts for antenna, antenna cable, and receiver delays. This delay constant is entered into the system software prior to shipment. Users are instructed not to change the delay constant, the antenna cable, or the antenna, as making any of these changes would invalidate the calibration.

**Fig. 4:**
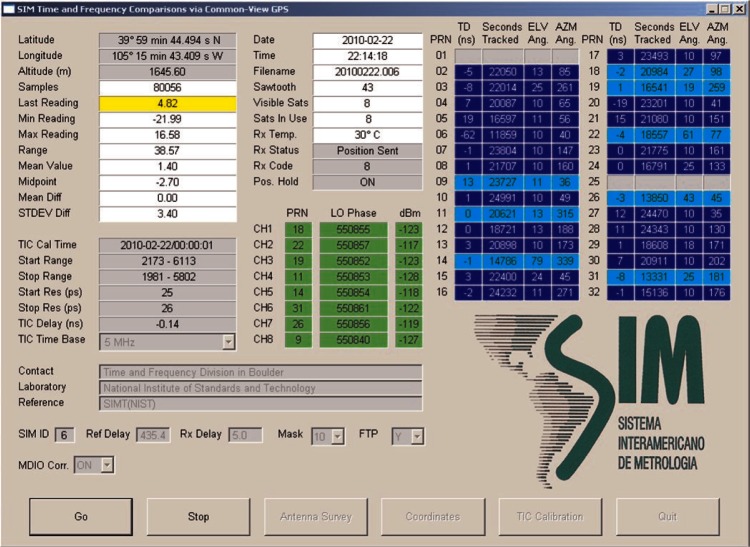
The SIM measurement system display.

The SIM measurement system consists of an industrial rack-mount computer that contains a time interval counter with single shot resolution of less than 0.1 ns, and an eight-channel GPS receiver. The display (Fig. 4) provides information about the GPS satellites being tracked and some statistics related to the measurements. The receiver is connected to an aperture coupled slot array antenna designed to mitigate the reception of multipath signals. This “pinwheel” type antenna is smaller and lighter than a choke ring antenna, but has been shown to reject multipath signals equally as well [8, 9].

The SIM system accepts either a 5 MHz or 10 MHz signal as the counter’s external time base, and a one pulse per second (1 pps) signal from the local time standard. The time difference between GPS and the local standard is measured every second, and both one-minute and 10-minute averages are recorded for as many as eight satellites. The 10-minute data files are the files transmitted via the Internet. These files include a header with the current system settings, followed by a 32 × 144 matrix containing the time measurements. The 32 column numbers match the pseudo-random noise (PRN) codes of the GPS satellites. The 144 rows represent the number of 10-minute segments in one day. This data format is unique to the SIMTN and incompatible with the Consultative GPS and GLONASS Time Transfer Sub-committee (CGGTTS) format used by the BIPM [10]. However, software that converts SIM data to the CGGTTS format has been developed to assist NMIs that need this capability. The native SIM format collects about 23 % more data than the CGGTTS multi-channel format, as shown in Table 1.

## 4. Near Real-Time Reporting of Results

A shortcoming of the common-view technique is that the results are sometimes not known until long after the measurements are made. This is because the data collected at both sites have to reside in one place before performing the subtraction shown in equations 2 and 3. The SIMTN solves this problem by transferring and processing data “on the fly.” Each system transfers its collected data via the Internet. Custom file transfer protocol (FTP) software installed on each SIM system transfers data every 10 minutes to servers located at CENAM, NRC, and NIST. This scheme stores copies of the SIMTN data in three different countries for redundancy.

The three SIMTN servers host identical software that processes common-view data whenever a request is received from a user. The measurement results can be viewed with any web browser by accessing any of the three servers. No special software is needed and no training is required. All three servers are linked from the web site of the SIM Time and Frequency Metrology Working Group at http://tf.nist.gov/sim. Each server displays a real-time grid that shows the most recent time differences between SIM NMIs. The grids receive new data every 10 minutes, and refresh every five minutes. If a user clicks on a time difference value displayed on the grid, a phase plot of the comparison for the current day will appear in their web browser. The phase plots can be adjusted to include up to 200 days of data. The results are also graphed as either one-hour or one-day averages and the TDEV and Allan deviation (ADEV) values for the selected data are automatically displayed. In addition to the graphs, 10-minute, one-hour, or one-day averages can be viewed in tabular form and copied to a spreadsheet for further analysis.

The real-time measurements allow all SIMTN participants to instantly compare their time standards to each other. This benefits all SIM NMIs, including those that already participate in the BIPM key comparisons and contribute to the computation of UTC. The UTC contributors can now check the performance of their standard without waiting for the key comparison results in the BIPM’s monthly *Circular-T* [11] report, which includes data that are typically from two to seven weeks old when published. Another advantage of the regional comparison is that data are reported every 10 minutes for the SIMTN, as opposed to every five days in the case of the *Circular-T*. This makes it much easier to identify short-term fluctuations and solve measurement problems. It seems likely that the BIPM key comparison results will eventually be processed in near real-time.

## 5. SIMTN Participants

**Fig. 5:**
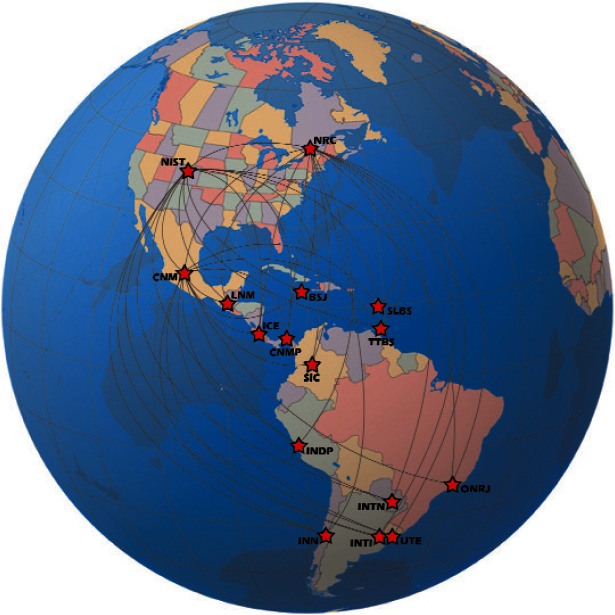
Map of the SIMTN.

As of late 2010, NMIs in 15 different nations are participating in the SIMTN. A measurement system has been shipped to the 16th nation (Chile), and they are expected to begin contributions soon. The current participants are listed in Table 2 and a map is provided in Fig. 5. We anticipate that other SIM NMIs will establish time and frequency laboratories, that additional requests to join the network will be received, and that the network will continue to expand. Excluding labor, the entire network in its present state (including 16 measurement systems, three servers, and shipping expenses) has cost slightly more than $100 000 USD, a modest price for such a major undertaking.

Table 2 also lists the type of national time and frequency standard maintained by each SIMTN participant. Four SIM NMIs operate time scales consisting of an ensemble of cesium oscillators and/or hydrogen masers: CENAM, NIST, NRC, and ONRJ. The other SIM NMIs maintain either a cesium oscillator, a rubidium oscillator, or a GPS disciplined oscillator as their primary standard. Some of the participants are maintaining a time and frequency standard for the first time. As more experience is gained, we expect SIM NMIs to upgrade their standards as resources become available, with some progressing from a rubidium oscillator to a cesium, and then eventually obtaining the multiple cesium oscillators needed to build an ensemble time scale. This progression has already begun. At least three laboratories have upgraded their standards since joining the SIMTN, including SIC in Colombia, INTI in Argentina, and ICE in Costa Rica.

## 6. Measurement Uncertainties

Estimating the uncertainties of the SIM network measurements involves use of both the Type A and Type B methods to evaluate uncertainties, as described in the ISO standard [17]. Uncertainties are combined with the root sum of squares method, where *k* is the coverage factor:

(4) $$U_{c}=k\sqrt{U_{A}^{2}+U_{B}^{2}}.$$

Time transfer noise is evaluated with the Type A method. We use TDEV at τ = 1 day, which is an established metric for estimating time transfer noise when the dominant noise type is white phase noise or flicker phase noise. For most SIMTN comparisons, TDEV at τ = 1 day is less than 3 ns, and is sometimes less than 1 ns for comparisons between NMIs with ensemble time scales. The time deviation should not exceed 5 ns if both laboratories involved in the comparison have either a cesium oscillator or a time scale (for comparisons involving rubidium oscillators, TDEV will be dominated by clock noise and can be much larger).

Seven other contributors to the uncertainty are evaluated with the Type B method. These uncertainties can potentially introduce systematic errors in the mean time offset between SIM standards. The uncertainties evaluated with the Type B method are discussed below and summarized in Table 3.

### 6.1 *U_B_*, Calibration

**Fig. 6:**
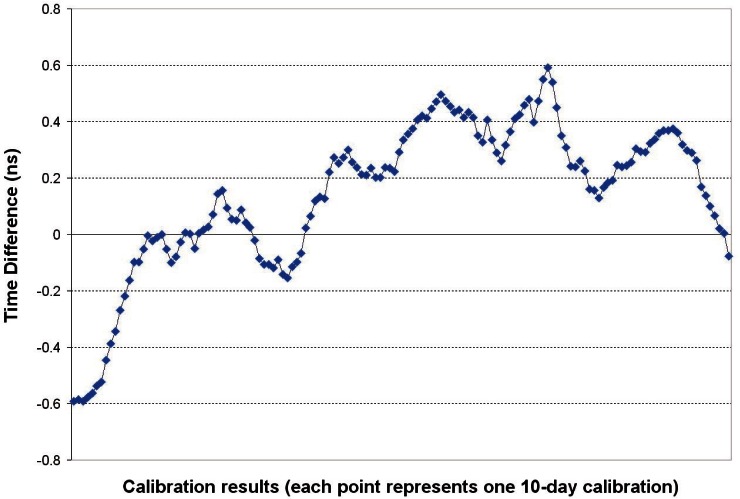
Delay variation during 141 consecutive 10-day common-clock calibrations.

The 10-day common-clock calibrations of SIM units performed at NIST in Boulder, Colorado produce a receiver delay estimate, D_Rx_, that is stored in the configuration file of each unit prior to shipment. These calibrations are typically stable to about 0.2 ns (TDEV at τ = 1 day) and have good repeatability. This is illustrated in Fig. 6, which shows results from a unit that was measured in common-clock mode for a 150-day period ending on February 15, 2010, the equivalent of 141 consecutive 10-day calibrations. During this interval, the peak-to-peak variation in the calibration results was 1.2 ns. Of course, larger variations can occur due to a variety of factors. Because the system will be operated in an environment different from that of the calibration site, we estimate that the calibration can contribute a measurement uncertainty of as large as 4 ns, with 2 ns perhaps being typical.

### 6.2 *U_B_*, Coordinates

The SIM NMIs are required to obtain GPS antenna coordinates prior to starting the measurements. If precise antenna coordinates are not available, the SIM system can survey the position of its antenna by averaging position fixes for 24 hours. This method can typically determine horizontal position (latitude and longitude) to within less than 20 cm. However, the self survey usually does a poor job of determining vertical position (elevation). Elevation errors can be as large as 10 m and can contribute timing uncertainties as large as 25 ns. For this reason, elevation is often obtained through an independent survey, typically by use of a dual frequency geodetic GPS receiver. Most SIMTN participants have been able to obtain both their horizontal and vertical coordinates to within 1 m, so the uncertainty due to antenna coordinates is typically less than 3 ns.

### 6.3 *U_B_*, Environment

GPS receiver, antenna, and antenna cable delays change as a function of temperature and other environmental factors. The SIM GPS receiver is more sensitive to temperature changes than either the antenna or antenna cable. Its temperature is not controlled, but is typically just a few degrees Celsius higher than the laboratory temperature, with a similar range. If sudden changes in laboratory temperature occur, the receiver delay can change by several nanoseconds, usually returning to its previous delay when the temperature returns to normal. Smaller delay changes can gradually occur for reasons that are not well understood, but that could be due to fluctuations in power supply voltages, vibration, or humidity.

The GPS antenna and part of the cable are outdoors, and are subjected to daily and seasonal variations in temperature. For example, the annual outdoor temperature range at NIST can exceed 60 °C. Even with such a wide range of temperature, the actual changes in the electrical delay of the cable are insignificant, but they can potentially cause the receiver tracking point to change and introduce phase steps in the data. The SIM systems reduce this possibility by using high quality antenna cables with low temperature coefficients.

Determining the source of a delay change can be difficult, and experience has shown that small delay changes due to environmental effects are inevitable, no matter how tightly the laboratory temperature is controlled. This problem is perhaps accentuated by the inexpensive hardware used to construct the SIM systems. We estimate this uncertainty to typically be about 3 ns, perhaps reduced to about 2.5 ns in a laboratory with tight temperature control.

### 6.4 *U_B_*, Multipath

Uncertainty due to multipath is contributed by GPS signals that are reflected from surfaces near the antenna. These reflected signals can interfere with, or be mistaken for, the signals that travel a straight line path from the satellite, resulting in delay changes. When possible, antennas are mounted in areas with a clear, unobstructed view of the sky on all sides, and the antenna itself was designed to mitigate multipath [8, 9]. This typically limits the uncertainty introduced by multipath to about 2 ns, but some types of multipath are difficult to avoid, and errors are large as 5 ns can occur in some instances.

### 6.5 *U_B_*, Ionosphere

The SIM systems apply the modeled ionospheric (MDIO) corrections broadcast from the GPS satellites to the measurements in real-time, and do not apply post-processed measured ionospheric (MSIO) corrections. Of course, ionospheric conditions are not identical at both sites (particularly when it is dark at one site and daylight at the other), and the use of locally generated MSIO corrections would provide better accuracy. The difference between the MDIO and MSIO corrections introduces time errors that generally increase as a function of the length of the baseline. For the 8623.5 km baseline between NIST and ONRJ, this uncertainty was estimated as 3.2 ns [6]. It should typically be about 2 ns for most SIM baselines, and less than that for comparisons between NMIs located in neighboring countries. For example, the baseline between Uruguay and Argentina is only 215.3 km.

### 6.6 *U_B_*, Reference Delay

Each NMI is responsible for measuring the reference delay, or DREF, and entering this value into the system software. The reference delay represents the delay from the NMI’s time standard to the end of the cable that connects to the SIM system. This measurement is normally made with a time interval counter and typically contributes an uncertainty of about 1 ns.

### 6.7 *U_B_*, Resolution

The SIM software limits the resolution of the entered delay values to 0.1 ns, which is roughly equivalent to the single-shot resolution of the time interval counter. This contributes an insignificant resolution uncertainty of 0.05 ns.

### 6.8 *U_C_*, Combined Uncertainty

Table 3 shows the “best case,” “worst case,” and “typical” uncertainties of the SIMTN comparisons. The “worst case” uncertainty can be avoided with a reasonably good survey of the GPS antenna. It is unlikely that all of the uncertainty components can be controlled at the “best case” level, but the “typical” combined uncertainty (*k* = 2) of 11.8 ns is achievable for most SIMTN comparisons.

## 7. Measurement Results

**Fig. 7:**
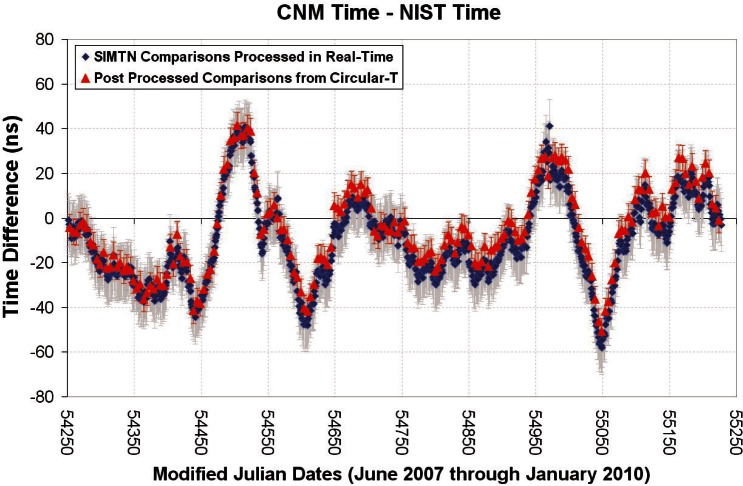
32-month comparison between Mexico and the United States.

In cases where SIMTN members also participate in the BIPM key comparisons, the results of the two measurements can be compared. The two measurements are made independently and utilize different GPS receivers, measurement hardware, and processing methods, but they agree within their stated measurement uncertainties, with considerable overlap in the coverage areas. For example, Fig. 7 shows the results of comparisons between the ensemble time scales of CENAM and NIST for the 32-month period beginning June 1, 2007 and ending January 31, 2010. The SIMTN values (one-day averages) have gray error bars showing an estimated uncertainty (*k* = 2) of 12 ns. The BIPM values are reported at five-day intervals and have red error bars that reflect the larger of the uncertainties (5.7 ns) reported on the *Circular-T* for the two NMIs (BIPM uncertainties are reported as *k* = 1). Note that the absolute time difference between NIST and CENAM never exceeded 60 ns during the entire comparison.

**Fig. 8:**
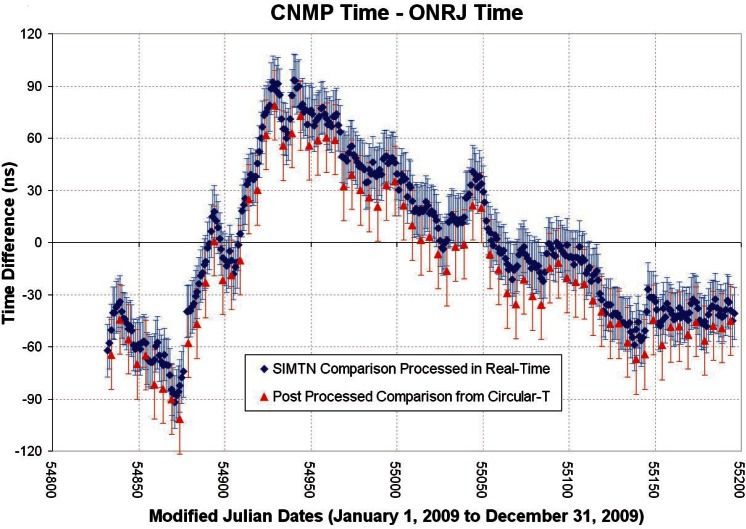
One year comparison between Panama and Brazil.

Figure 8 shows the SIMTN and BIPM *Circular-T* results for a comparison between CENAMEP and ONRJ for the entire year of 2009. The blue error bars of the SIMTN show the coverage area of the estimated *k* = 2 uncertainty of 15 ns. The BIPM values have red error bars that reflect the larger of the uncertainties (20 ns) reported on the *Circular-T* for the two NMIs. As in Fig. 7, there is considerable overlap between the uncertainties of the two independent measurements.

**Fig. 9:**
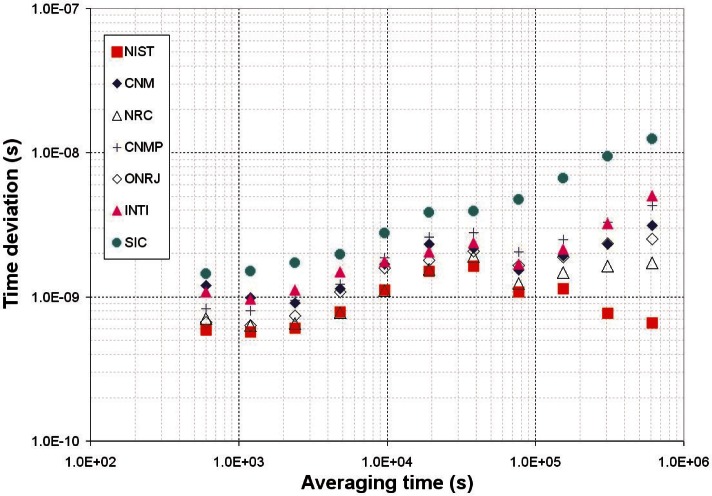
Timing stability of SIMTN time standards relative to GPS.

**Fig. 10:**
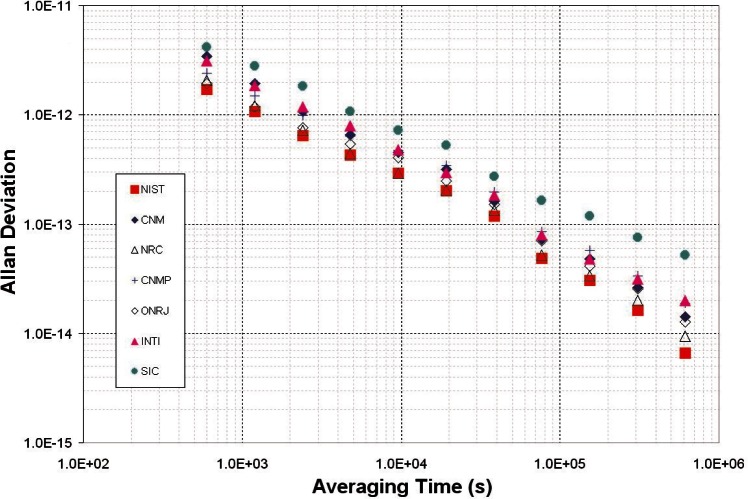
Frequency stability of SIMTN time standards relative to GPS.

The stability of the SIM time standards can be estimated by comparing them to the GPS data collected by each NMI. Each SIM system contains identical GPS hardware that was calibrated in the exact same way, so GPS serves as a convenient and independent standard of comparison. Figure 9 shows the time stability (TDEV) of seven SIMTN participants during the last six months of 2009 for averaging periods ranging from 10 minutes to about one week. Figure 10 shows the frequency stability (ADEV) for the same participants over the same interval.

The SIMTN has undoubtedly improved time and frequency coordination within the SIM region. Table 4 shows the average and maximum time offsets (rounded to the nearest nanosecond) and the average frequency offset (rounded to the nearest part in 10^15^) between the nine SIMTN participants who operated either cesium oscillators or ensemble time scales during the last six months of 2009. Note that seven of the nine NMIs kept average time within ± 21 ns of each other during the six month period. The frequency differences between most labs during the six-month interval were less than 5 × 10^−15^. These results indicate that time and frequency standards are now kept in relatively close agreement throughout the SIM region.

## 8. Benefits to the SIM Region

The SIM time and frequency working group and the SIMTN have improved time metrology throughout the Americas in several ways. The SIM effort has led to the establishment of a SIM time scale, improved quality systems and calibration and measurement capabilities (CMCs), and improved educational and collaboration opportunities. These benefits are briefly discussed in this section.

The quest for even more rigorous time and frequency coordination than that shown in Table 4 has led to the establishment of a SIM Time Scale (SIMT). Work on the algorithms for SIMT began at CENAM in late 2008. The SIMT system accepts the real-time inputs from each SIM laboratory that operates a cesium standard or an ensemble time scale, and generates a composite time scale in real-time based on the weighted average of each contributor. Results are updated hourly and published on-line at http://tf.nist.gov/sim. The generation of a regional time scale makes it possible for SIMTN participants to compare their standards not only to each other, but also to SIMT [18].

An important goal of the SIM effort is to have all NMIs develop quality systems and to submit their calibration and measurement capabilities (CMCs) to the BIPM Key Comparison Database (KCDB) so that their calibrations can be internationally recognized. When the SIMTN was first established, none of the SIM NMIs were included in the KCDB for time and frequency. The first to be included was CENAMEP in August 2006. As of late 2010, five SIM timing laboratories (CENAM, CENAMEP, ONRJ, NIST, and NRC) are among the 42 timing laboratories included in the KCDB, and several others are working on their submissions. More work remains to be done in this area, but considerable progress has been made.

The value of metrology education cannot be overstated. The staff members at small and recently established NMIs obviously benefit from the experience of their colleagues at well established laboratories, but all NMIs have unique experiences that they can share with the others. For example, in many cases, the smaller NMIs perform more calibrations and have more direct experience working with industry. To further the cause of metrology education, SIM has conducted three four-day time and frequency training classes, with each well attended by metrologists from both NMIs and industry. The first was held in Asunción, Paraguay in December 2005, the second was in Buenos Aires, Argentina in February 2008, and the third in Lima, Peru in March 2010. The training effort goes on continuously through emails, phone conversations, and occasional laboratory visits, and the communication between SIM NMIs has been excellent. This communication has led to an increase in the number of scientific and calibration related collaborations between SIM laboratories. In turn, the increase in collaborations should lead to more rapid scientific advances and more efficient operations.

## 9. Benefits to Individual NMIs

Participation in the SIMTN has provided benefits to individual NMIs, helping them to gain status as the official timekeeper for their country, to better support the industrial time and frequency requirements of their country, and to develop new time and frequency services. These benefits are briefly discussed in this section.

A goal of many SIM NMIs is to gain recognition as the official source of time for their country, an important responsibility. Some SIMTN participants gained recognition as official timekeepers long ago, for example, NRC has been the official timekeeper for Canada since 1970, by order of the Canadian Parliament. However, new NMIs must first establish name recognition within their countries, demonstrate the ability to maintain an internationally recognized time standard, and then begin the legislative process required to obtain official timekeeper status. This goal was accomplished by INTN in Paraguay by presidential decree in December 2009, largely due to the presence of the SIMTN. SIC had fulfilled the responsibility of being Colombia’s official timekeeper since 1992, but received legal confirmation of this function (decree 3523) in 2009, thanks in part to the SIMTN. With the help of the SIMTN, UTE is now working on an agreement to audit the agency responsible for the official time in Uruguay, and SNM is collaborating with the Peruvian military to provide the official time for Peru. A number of other SIMTN participants, including BSJ, CENAMEP, ICE, and TTBS are now working towards similar goals and expect to be successful.

As noted previously, four SIM NMIs operate ensemble time scales. These four represent a significant percentage (perhaps 30 % to 40 %) of the ensemble time scales that currently exist at NMIs worldwide. At least three other SIMTN participants (BSJ, CENAMEP, and SIC) have announced plans to build ensemble time scales in the future.

The SIMTN has also helped with the development of new time broadcast and calibration services throughout the SIM region. New network time protocol (NTP) servers have been added by CENAMEP, ICE, and SNM, and are planned at INTN and elsewhere. Web clocks, a convenient way to distribute time-of-day to the general public, are now operated by seven SIMTN participants, and several other laboratories have announced plans to develop them. NIST launched its Time Measurement and Analysis Service (TMAS), a remote calibration service intended for metrology laboratories and research facilities, by utilizing technology and experience gained from the SIMTN [19] and a similar service is operated at CENAM. Experience gained from the SIMTN also allowed CENAM and NIST to collaborate on a project to synchronize the clocks in the TELMEX communications network in Mexico to CENAM time. TELMEX is the largest telephone provider in Mexico and serves many millions of customers. Their telephone network includes eight cesium primary reference clocks, located in four different cities in Mexico. The goal of the project was to continuously compare the eight cesium clocks to the national time standard in Mexico. The goal was accomplished by building a time network for TELMEX that is similar to the SIMTN. ICE is doing similar work, and is monitoring clocks in the telecommunications synchronizing network in Costa Rica.

## 10. Summary and Conclusion

The SIMTN is an excellent example of how a RMO can improve the status, recognition, and capabilities of the NMIs within its region. It has not only accomplished its basic objective of providing NMIs with a convenient way to establish traceability to the SI, but has also provided other benefits that have enhanced time and frequency metrology throughout the Americas. After beginning operation in three nations in May 2005, the SIMTN now traverses across 16 nations, with more SIM nations expected to join in the future. This rapid expansion, along with the improved capabilities of SIM timing laboratories, clearly indicates that contributions from the Americas to the world's timekeeping community are on the rise. We expect this trend to continue for many years.

## Figures and Tables

**Table 1:** Comparison of common-view data formats

Data Format	Daily Tracks	Track Length (min.)	Satellites Tracked	Total Minutes Tracked
CGGTTS Single-channel	48	13	1	624
CGGTTS Multi-channel	90	13	8 typical	9 360
SIMTN	144	10	8 max	11 520

**Table 2:** Current and Future SIM Network Members

Country	NMI	Month of First Participation	National Standard
Argentina	INTI	January 2008	Cesium
Brazil	ONRJ	May 2007	Time Scale [6, 12]
Canada	NRC	June 2005	Time Scale [13]
Chile	INN	December 2010	Rubidium
Colombia	SIC	May 2007	Cesium
Costa Rica	ICE	March 2007	Cesium
Guatemala	LNM	November 2009	GPSDO
Jamaica	BSJ	January 2008	Cesium
Mexico	CENAM or CNM	May 2005	Time Scale [14]
Panama	CENAMEP or CNMP	December 2005	Cesium
Paraguay	INTN	February 2009	Rubidium
Peru	SNM	September 2009	Rubidium
St. Lucia	SLBS	June 2010	Rubidium
Trinidad / Tobago	TTBS	November 2009	GPSDO
United States	NIST	May 2005	Time Scale [15]
Uruguay	UTE	January 2009	Disciplined Rubidium [16]

**Table 3:** Measurement Uncertainties (nanoseconds)

Uncertainty Component	Best Case	Worst Case	Typical
U_A_, TDEV, τ = 1 d	0.7	5	2
U_B_, Calibration	1	4	2
U_B_, Coordinates	1	25	3
U_B_, Environment	2.5	4	3
U_B_, Multipath	1.5	5	2
U_B_, Ionosphere	1	3.5	2
U_B_, Ref. Delay	0.5	2	1
U_B_, Resolution	0.05	0.05	0.05
U_C_, *k* = 2	7.0	53.8	11.8

**Table 4:** Time and Frequency Differences between SIM NMIs (July 1 to December 31, 2009)

Maximum Time Difference (ns)	NIST	CNM	NRC	CNMP	ONRJ	ICE	SIC	INTI	BSJ
NIST		57	−95	38	25	−1066	−6	52	51
CENAM	−57		−111	−81	−38	−1081	−74	68	−80
NRC	95	111		122	93	−997	118	133	138
CENAMEP	−38	81	−122		−59	−1088	−88	57	56
ONRJ	−25	−38	−93	59		−1084	−46	61	68
ICE	1066	1081	997	1088	1084		1032	1072	1098
SIC	56	74	−118	88	46	−1032		79	100
INTI	−52	−68	−133	−57	−61	−1072	−79		−71
BSJ	−51	80	−138	−56	−68	−1098	−100	71	

Average Time Difference (ns)	NIST	CNM	NRC	CNMP	ONRJ	ICE	SIC	INTI	BSJ

NIST		10	−73	13	<1	−480	−8	15	11
CENAM	−10		−82	4	−9	−489	−18	3	<1
NRC	73	82		86	71	−407	65	86	84
CENAMEP	−13	−4	−86		−17	−492	−21	−7	−2
ONRJ	<1	9	−71	17		−476	−6	11	13
ICE	480	489	407	492	476		456	487	464
SIC	8	18	−65	21	6	−456		16	18
INTI	−15	−3	−86	7	−11	−487	−16		4
BSJ	−11	<1	−84	2	−13	−464	−18	−4	

Average Frequency Difference (× 10^−15^)	NIST	CNM	NRC	CNMP	ONRJ	ICE	SIC	INTI	BSJ

NIST		−4	−2	3	−2	15	−1	<1	1
CENAM	4		2	6	2	19	2	4	5
NRC	2	−2		5	<1	17	<1	2	4
CENAMEP	−3	−6	−5		−5	13	−4	−3	−1
ONRJ	2	−2	<1	5		17	<1	2	4
ICE	−15	−19	−17	−13	−17		−21	−15	−28
SIC	1	−2	<1	4	<1	21		2	3
INTI	<1	−4	−2	3	−2	15	−2		2
BSJ	−1	−5	−4	1	−4	28	−3	−2	
